# Investigating intrinsic and evoked activities in cultured neuronal networks by dimensional reduction techniques and high-density MEAs

**DOI:** 10.1186/1471-2202-16-S1-P13

**Published:** 2015-12-18

**Authors:** Thierry Nieus, Stefano Di Marco, Alessandro Maccione, Hayder Amin, Luca Berdondini

**Affiliations:** 1 Neuroscience Brain Technology Istituto Italiano di Tecnologia, via Morego 30, 16163 Genoa, Italy

## 

High density microelectrode arrays (MEAs) provide extracellular recordings from thousands of closely spaced electrodes and with sub-millisecond resolution. These devices offer thus novel capabilities to investigate the interplay between ongoing and evoked electrophysiological signaling within networks in-vitro. However, to effectively take advantage from the spatiotemporal resolution of these MEAs, adapted analysis tools are needed. Here we report on our recent advancements toward this goal. A novel high density MEA with on-chip stimulating electrodes was used to record from 4096 electrodes and electrical stimulation was delivered alternatively through one of the 16 equally spaced electrodes on hippocampal primary cultures derived from mouse. The evoked activities propagated reliably across the network and were specific to the stimulating electrode [1]. Moreover, from the second week in vitro cell cultures also displayed synchronous like events (called synchronous burst events, SBEs) that propagated similarly to the evoked activities across the entire network. These propagations might be informative of the underlying network connectivity and their classification based on the spatiotemporal patterns might elucidate the network's organization and its ongoing dynamic. Former attempts, in classifying SBEs, considered simplified descriptors of neural activity (e.g. center activity trajectory [2]). Here, we have adopted a more rigorous approach by applying dimensional reduction techniques (PCA) that take advantage of the redundancy and of the sparseness of multi-unit recordings. We found that a large fraction (i.e. >50%) of the variance of the network events (either evoked or spontaneous) was explained by as few as 3 principal components (PCs). By increasing the number of PCs (i.e. ~10) up to 80% of the total variance of the network events could be explained. Thus, the PCA approach constitutes an effective methodology to represent the spontaneous/evoked events in a lower dimensional space. As a consequence the principled PCA methodology we developed improved the clustering of the network events respect to existing methodologies [2] in different experimental settings (e.g. treatment with chemical compounds). Finally, the PCA clustering approach also allowed to infer on state dependent processing phenomena occurring in these networks.
